# The Effects of Intermittent Trunk Flexion With and Without Support on Sitting Balance in Young Adults

**DOI:** 10.3389/fnhum.2022.868153

**Published:** 2022-03-29

**Authors:** Matej Voglar, Žiga Kozinc, Idsart Kingma, Jaap H. van Dieën, Nejc Šarabon

**Affiliations:** ^1^Faculty of Health Sciences, University of Primorska, Izola, Slovenia; ^2^Andrej Marušič Institute, University of Primorska, Koper, Slovenia; ^3^Department of Human Movement Sciences, Faculty of Behavioural and Movement Sciences, Amsterdam Movement Sciences, Vrije Universiteit, Amsterdam, Netherlands; ^4^InnoRenew CoE, Human Health Department, Izola, Slovenia; ^5^Laboratory for Motor Control and Motor Behavior, S2P, Science to Practice, Ltd., Ljubljana, Slovenia

**Keywords:** trunk stiffness, reflex gain, postural control, postural stability, spine stability

## Abstract

Prolonged trunk flexion is known to affect passive and active stabilization of the trunk. Previous studies have evaluated changes in spinal range of motion, muscle activity and reflex behavior induced by prolonged trunk flexion, whereas the effect on sitting postural control is vastly underexplored. In this study, we compared the effects of supported and unsupported intermittent trunk flexion on center of pressure (CoP) motion during sitting on an unstable seat. Participants (*n* = 21; 11 males, 23.2 ± 2.0 years; 10 females, age 24.3 ± 4.0) were exposed to 1-h intermittent (60-s sets with 30 s of rest) trunk flexion (80% of the maximal range of motion) and CoP root mean square distance, velocity and frequency before and after the exposure were assessed. Contrary to our hypothesis, there were no main effects of exposure (pre. vs. post flexion protocol; *p* = 0.128–0.709), no main effects of condition (supported vs. unsupported; *p* = 0.134–0.931), and no interaction between exposure and condition (*p* = 0.163–0.912). Our results indicate that prolonged intermittent flexion does not induce any changes in CoP motion during a seated balance task, regardless of the presence of a trunk support during prolonged intermittent flexion. This suggests a successful compensation of decreased passive stiffness by increased reflex activity.

## Introduction

In the working-age population, between 20 and 40% of persons suffer from low back pain (LBP) annually (Hoy et al., 2012). Physically demanding professions that include manual material handling and working in awkward postures have been considered to increase the risk of LBP (Heneweer et al., 2011; Griffith et al., 2012; Fatoye et al., 2019), although recent publications report no clear consensus regarding the causality between posture and presence of LBP symptoms (Swain et al., 2020). A strong case for an association between LBP risk and working with twisted or bent trunk has been reported in a systematic review, although cause-and-effect relationship could not be conclusively confirmed (Wai et al., 2010). Prolonged trunk flexion has been shown to alter both passive mechanical properties of the spinal column as well as active control of spinal stability (Sánchez-Zuriaga et al., 2010; Bazrgari et al., 2011; Hendershot et al., 2011; Howarth et al., 2013; Voglar et al., 2016), which is believed to increase the risk of LBP.

The effect of prolonged trunk flexion on spinal stability has been mostly evaluated by measuring maximal lumbar flexion range of motion, which is used as an indicator of the creep deformation and consequent decrease in passive trunk stiffness. Creep deformation after prolonged trunk flexion is consistently shown (McGill and Brown, 1992; Rogers and Granata, 2006; Sánchez-Zuriaga et al., 2010), and probably follows a dose-response relationship (Hendershot et al., 2011; Muslim et al., 2013). On the other hand, the effects of prolonged trunk flexion on intrinsic stiffness (i.e., stiffness due to passive tissues and pre-activated muscles) and reflexive trunk stiffness (i.e., stiffness due to feedback activation of muscles) are less clear. For instance, Hendershot et al. (2011) reported decreased intrinsic trunk stiffness and increased reflex gains after 2 and 16 min of sustained trunk flexion. While the trunk stiffness rapidly returned to baseline levels, the reflex gains remained elevated at least 60 min. In contrast, another study by the same research group reported quick restoration of reflex gains, but slower recovery of intrinsic trunk stiffness following 10 min of sustained trunk flexion, although in males the reflex gains were again elevated 30 min after the onset of recovery period (Bazrgari et al., 2011). Finally, in two studies from another group, both increased and decreased reflex gais were found after exposure to spinal flexion (Granata et al., 2005b; Rogers and Granata, 2006).

In addition to analyzing discrete responses to external mechanical perturbations, evaluation of postural stability through center of pressure (CoP) movement quantification in a sitting posture has also been used to assess trunk stability (Van Dieën et al., 2012; Hendershot et al., 2013; Leban et al., 2017). Sitting postural sway has been shown to increase consistently throughout a simulated shift in crane operators (Leban et al., 2017), as well as throughout long-distance shifts in bus riders (Arippa et al., 2021). These observations could be explained by muscle fatigue, which has consistently been shown to deteriorate standing postural sway (Paillard, 2012). Specifically, trunk extensor muscle fatigue induced by crane operator workload could translate to increased sway in sitting position, as these muscles are paramount for sitting postural control (Curtis et al., 2015). On the other hand, studies investigating electromyographic (EMG) muscle responses indicate that reflexive trunk stability after an exposure to flexion is impaired primarily due to the creep deformation of soft tissues, and not due to muscle fatigue (Sánchez-Zuriaga et al., 2010). Assessing CoP behavior during sitting seems as a promising tool to investigate changes in trunk stability.

To the best of our knowledge, only one study has investigated the effects of exposure to flexion on CoP behavior during sitting (Hendershot et al., 2013). This study showed that both creep deformation and CoP movements increased in a dose-response fashion (2-, 4-, and 10-min exposures were used), while there were no significant differences in recovery patterns between different exposures, with 10 min being sufficient to restore CoP behavior to baseline. It remains to be determined how CoP behavior in seated position is affected by longer flexion periods, and whether the provision of trunk support can attenuate the decrements in sitting postural control. In this paper, we report the results pertaining to seated postural stability, obtained in a larger experiment (Voglar et al., 2016) that was conducted to compare the effects of supported and unsupported prolonged intermittent flexion exposure on trunk stability. The support in the former condition was provided as a padded bar, on which the participants leaned with their chests and shoulders. Total trunk stiffness increased after unsupported flexion only, while reflex gains increased after both conditions. A larger increase in lumbar range of motion and reflex gains were noted following unsupported flexion in comparison to supported flexion (Voglar et al., 2016), which indicates a potentially beneficial effect of trunk support during working in flexed postures. The aim of this paper is to analyze the effects of exposure to prolonged intermittent flexion with and without support on sitting postural stability, assessed through CoP movement recording. Considering previous evidence (Hendershot et al., 2013; Voglar et al., 2016), we hypothesized that CoP movement amplitude and velocity will increase, with a concomitant decrease in CoP frequency, indicating impaired postural control after the exposure to intermittent flexion. We also hypothesized that the provision of support will eliminate or attenuate this effect.

## Materials and Methods

### Participants

Twenty-one young participants were included in the present study [11 males, (age 23.2 ± 2.0 years, height 182.3 ± 6.2 cm, and body mass 73.9 ± 8.2 kg) and 10 females, (age 24.3 ± 4.0) years, height 168.3 ± 7.2 cm, and body mass 62.1 ± 9.0 kg)]. Participants who reported LBP within the last 6 months, or any history of LBP that impaired their physical activity for at least 1 day, were excluded. Moreover, participants with any known sensory or neuromuscular pathologies were also excluded. The experimental protocol was approved by the Ethics committee for Movement Sciences at the Vrije Universiteit, Amsterdam (approval number: ECB 2015–18). All subjects were required to sign an informed consent statement prior to the experiment. The study was conducted in accordance with the Helsinki Declaration.

### Pilot Experiment

The purpose of the pilot experiment was to establish the number of trials of seated balance assessment needed to obtain reliable performance. For this purpose, 12 participants (seven males, five females; age: 28.2 ± 5.8) performed a two-session experiment, with 10 repetitions of the seated balance task (see Section Sitting Balance Assessment for details). The reliability in a pilot experiment was assessed with single-measures, two-way random model intra-class correlation coefficients (ICCs) for absolute agreement. Reliability was considered as excellent when ICC was > 0.90, and good when ICC > 0.75 (Koo and Li, 2016). Values below 0.75 were considered as unacceptable. None of the participants in the pilot experiment participated in the main experiment.

### Study Design

**FIGURE 1:**
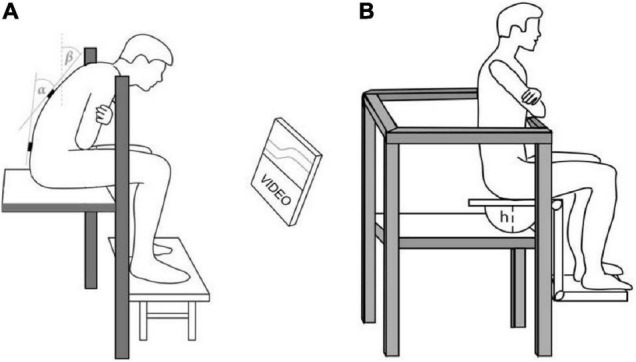
**(A)** During intermitant flexion exposure participants obtained ∼80% of their available lumbar flexion (α) and 35° of trunk inclination (β) using real-time visual feedback. Additionally in the supported condition a padded bar was placed in front of the participant at appropriate height in order to lean on it while obtaining the required trunk position. **(B)** Postural control assessment on a custom-built chair with a hemi-sphere attached below the seat surface [height (h) = 18 cm].

The study protocol consisted of two visits, each consisting of one of the two exposure conditions: supported flexion (SF) and unsupported flexion (USF) (see Section Intermittent Trunk Flexion Task; Figure 1A). The experiment also involved the assessment of trunk reflex gains and range of motion. Sitting balance was always measured after trunk reflex gains and range of motion assessments. The details regarding the full procedure are available in our previous paper (Voglar et al., 2016). Before the baseline measurements, the participants performed a set of measurements followed by siting for 30 min on an office chair with their backs supported against the backrest. This was done to provide a washout period, avoiding any effects of previous activities of the participants. The measurements included assessment of postural control in a sitting posture via CoP movement analysis (see Section Sitting Balance Assessment; Figure 1B). After the baseline measurements, participants were exposed to one of the two experimental conditions (SF and USF). The conditions were introduced on separate visits, in a quasi-randomized counterbalanced order, with a minimum of 4 days between visits. After the experimental condition, the sitting postural control assessment test was repeated. In all participants, less than 10 min passed between the end of the exposure and the assessment of postural control. During this time range of motion measurements and two perturbation trials were performed (Voglar et al., 2016).

### Intermittent Trunk Flexion Task

The participants were exposed to prolonged intermittent flexion, as described in detail in our previous study (Voglar et al., 2016). Briefly, the participant was seated on a raised platform with the feet supported and rails provided for safety (Figure 1A). Real-time feedback on lumbar flexion and trunk inclination was provided (see section Sitting Balance Assessment). Participants were reminded to adjust their position if they drifted more than 2° from the target position as checked by feedback on the computer screen. The target lumbar flexion angle was determined as 80% of the range of motion from erect stance to maximal forward flexion. To standardize loading and avoid that subject would obtain lumbar flexion by slumped sitting, trunk inclination and lumbar flexion were monitored. To determine the target posture, participants flexed forward until the trunk inclination reached 35°, and then adjusted the lumbar flexion position by tilting the pelvis until reaching 80% of lumbar flexion RoM. Target lumbar flexion percentage, inclination angle and exposure duration were adopted from one of the rare previous studies investigating effects of longer lasting (60 min) supported flexion on trunk neuromuscular control (Sánchez-Zuriaga et al., 2010). Both the target flexion percentage and inclination angle were slightly increased in comparison to the aforementioned study. This was done to increase the load on the trunk muscles during the unsupported condition, creating a greater contrast with the condition with trunk support. Intermittent trunk flexion was used to simulate the main characteristics of the harbor crane operator workload. The ratio between flexion and upright sitting was set based on our previous experiences and observation of crane operators work. Perceived discomfort or fatigue were not systematically assessed, but was frequently reported spontaneously during the unsupported condition, but not during the supported condition.

Muscle activity was tracked using surface EMG (REFA, TMSi, Netherlands), with the electrodes placed bilaterally over two segments of the erector spinae muscle (3 cm lateral to interspinous space between L4 and L3 and 6 cm lateral to the L2 spinous process). The details regarding EMG procedures are available in our previous report (Voglar et al., 2016). In case the participant presented with EMG silence of the back muscles due to the flexion relaxation phenomenon, the lumbar flexion angle was reduced until marked activation could be seen. The same lumbar flexion angle was used in both conditions. The flexion task was intermittent and consisted of 40 cycles including 1 min of target flexion level, followed by 30 s of upright active sitting, resulting in total duration of 60 min. Auditory cues were used to indicated the time to change position. In the unsupported flexion condition, a thin rope was placed horizontally to provide the participant with a mechanical orientation to indicate the required trunk inclination. Participants had their hands crossed across the chest and were touching the rope slightly with their shoulders. In the supported flexion the rope was replaced with a padded bar that provided passive support. The participants leaned on the pad with their chests and shoulders.

### Sitting Balance Assessment

Sitting postural control assessment involved the analysis of CoP movements whilst the participants were seated on a custom-built chair with a spherical surface attached below the seat surface (radius = 22 cm; height = 18 cm). The seat was placed on a platform where a force plate (KAP-E, AST, Germany) was installed and safety rails provided (Figure 1B). To control for task familiarization and ensure reliability, the participants performed six trials, each lasting 1-min, with 1-min breaks in between. The instruction was to stay as still as possible and reach for the rail only in case of losing balance. In case that balance was lost, the trial was repeated (one participant at baseline and one participant after the intermittent flexion). The force plate signal was sampled at 200 Hz and all CoP data were demeaned prior to further analyses. The postural control of the trunk was quantified by three variables (each calculated for anterior-posterior and medial-lateral direction) derived from CoP time series: root mean square (RMS) distance (mm), mean sway velocity (mm/s), and mean sway frequency (Hz), following the study by Larivière et al. (2013), which also showed high reliability of these measures specific for the sitting position.

### Lumbar Range of Motion

Pelvis and thorax orientations were estimated using two inertial measurement units (IMU) (Xsens Technologies X-bus, Enschede, Netherlands) positioned at the T12 and S2 level, directly on the spinous processes. Maximal lumbar flexion RoM was calculated as the difference in the inclination angles of the sensors in the sagittal plane. To achieve full lumbar flexion in standing position, the participants were instructed to maximally flex forward while keeping their knees slightly bent. Each participant performed two repetitions at baseline and after the exposure to the intermittent flexion protocol, and the highest value of the two repetitions was used for further analyses. The real-life feedback from the same two sensors was used to control the position of the spine during the intermittent flexion protocol (Section Study Design).

### Statistical Analysis

Statistical analyses were done with SPSS (version 25.0, SPSS Inc., Chicago, IL, United States). Descriptive statistics are reported as mean ± standard deviation. The normality of the data distribution was verified with Shapiro–Wilk tests. The inter-visit reliability of baseline values in the main experiment was assessed with ICC, as well as with 1-way repeated measures analysis of variance (ANOVA). Two-way ANOVA was used to analyze the effect of the exposure (pre. vs. post protocol) and the inclusion of support (supported vs. unsupported condition), as well as the interaction between the two main effects. Effect sizes were expressed as partial eta-squared (η^2^) and interpreted as trivial (<0.01), small (0.01–0.06), medium (0.06–0.14) and large (>0.14) (Bakeman, 2005). The threshold for statistical significance was set at α < 0.05.

## Results

### Pilot Reliability Experiment

**FIGURE 2:**
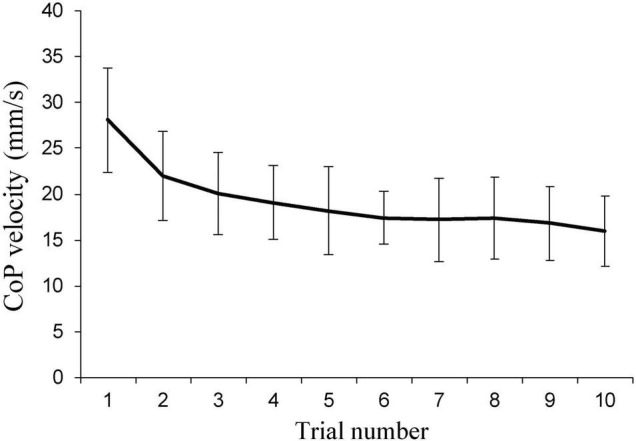
Within-session behavior of center of pressure (CoP) velocity in the pilot experiment (*n* = 12).

Our pilot experiments showed that 6 repetitions are needed to achieve stable sitting balance performance outcomes. The within-session reliability for CoP velocity was poor (ICC = 0.42) when considering repetitions 1–3, good when considering repetitions 2–4, 3–5, or 4–6 (ICC = 0.77–0.85), and excellent when considering repetitions 5–7 (ICC = 0.94). Considering even higher repetition numbers did not increase the ICC further. The inter-visit reliability for the average of repetitions 4–6 was also close to excellent (ICC = 0.89). Therefore, six repetitions were performed in the main experiment, and repetitions 4–6 were considered for further analyses. Figure 2 shows the behavior of CoP velocity across trials within the first session of the pilot experiment. Other variables showed very similar patterns (data not shown).

### Reliability of Baseline Measures in the Main Experiment

The analysis of variance showed no differences between sessions of baseline values for CoP RMS distance (*F* = 0.513–0.906; *p* = 0.352–0.482 over AP, ML and combined directions), CoP velocity (*F* = 0.396–0.958; *p* = 0.333–0.536), or CoP frequency (*F* = 0.015–0.222; *p* = 0.643–0.905). The ICC indicated moderate reliability for RMS outcomes (ICC = 0.61–0.75) and velocity outcomes (ICC = 0.64–0.72), but good reliability for frequency outcomes (ICC = 0.77–0.84).

### Effects of Intermittent Trunk Flexion on Sitting Balance

**FIGURE 3:**
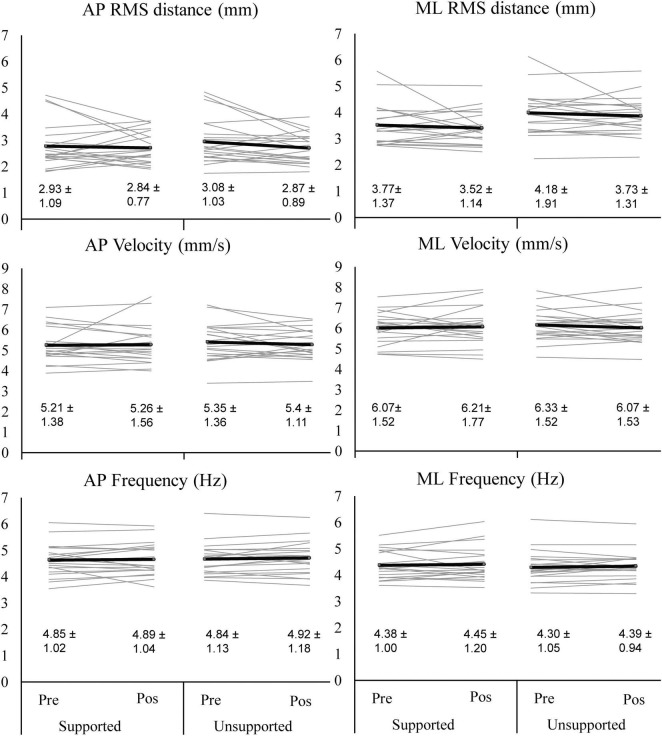
Center of pressure RMS (root mean square) distance, velocity and frequency before (Pre) and after (Pos) the exposure to supported and unsupported flexion. Gray lines indicate individual datapoints, and the black lines represent mean values. Note that combined antero-posterior (AP) and medio-lateral (ML) trajectory showed similar results and none of the effects were significant. The values below the lines are mean ± standard deviation.

Intermittent trunk flexion had no effect on CoP RMS distance, CoP velocity and CoP frequency in any direction (Figure 3). There were no main effects of exposure (pre. vs. post flexion protocol; *F* = 0.212–2.515; *p* = 0.128–0.709 over outcomes and directions), no main effects of condition (supported vs. unsupported; *F* = 0.003–2.443; *p* = 0.134–0.931), nor was there any exposure × condition interaction (*F* = 0.013–2.097; *p* = 0.163–0.912). All the associated effect sizes were small to medium for the effects of exposure (η^2^ = 0.01–0.11), trivial to medium for support condition (η^2^ = 0.00–0.11) and trivial to medium for the interaction (η^2^ = 0.00–0.10).

## Discussion

The purpose of this study was to assess the effects of supported and unsupported prolonged intermittent trunk flexion on postural control during sitting. Based on the previous studies reporting increases in CoP movements after much shorter exposures to flexion (Hendershot et al., 2013), we expected to observe an increase in CoP movements. In addition, we hypothesized that trunk support would attenuate or eliminate the effects of prolonged flexion in CoP movements, as we previously found that support attenuated or negated effects of lumbar flexion exposure on lumbar range of motion, admittance and reflex gains (Voglar et al., 2016). However, the hypotheses of the present paper were rejected, as the CoP movement was not affected by prolonged intermittent flexion, and there was no difference between supported and unsupported conditions. This was somewhat surprising, as we reported in the previous paper that the range motion was increased in both conditions, confirming the presence of viscoelastic deformation of passive tissues, which should decrease the stability of the trunk. It could be that the decrease in passive stiffness was successfully compensated by increase in reflex gains (Voglar et al., 2016). As reported in the previous paper (Voglar et al., 2016), the activation of erector spine (pars lumborum and pars iliocostalis) in response to forward directed force (60 N) was increased after the exposure to intermittent flexion and the increase in activation was higher following the unsupported condition. The increase in muscle activation could be a result of an increased neural drive to compensate for a reduced force production capacity of fatigued muscles and reduced intrinsic stiffness.

Contrary to our findings, Hendershot et al. (2013) reported significant increases in CoP RMS and velocity after 2–10 min of exposure to sustained flexion. Moreover, an increase in sitting postural sway was seen throughout a simulated crane operator shift (Leban et al., 2017). An important limitation of our study is the break (5–10 min) between the end of the flexion exposure and sitting balance assessment. It could be that this time was sufficient to restore CoP behavior to baseline. Another possible explanation for these discrepancies is the nature of the task, as we used intermittent flexion, whereas Hendershot et al. (2013) used sustained flexion. However, considering that changes induced by trunk flexion are reported to last longer than the exposure itself (McGill and Brown, 1992; Hendershot et al., 2011), we think it is unlikely that the breaks in our study were sufficient to eliminate all the effects. Another issue to consider is the range of motion, as we determined flexion position at 80% of full range of motion. The angle used in the flexion task is a significant determinant of the changes in trunk stiffness and reflex gain (Hendershot et al., 2011). Moreover, previous studies have also reported significant creep deformation and alteration of reflex behavior following the exposure to flexion at non-maximal ranges of motion (Sánchez-Zuriaga et al., 2010; Hendershot et al., 2011). Finally, the first three repetitions in our pilot experiment showed poor agreement (ICC = 0.42), indicating the need for sufficient familiarization before baseline assessment. Therefore, we used an extensive familiarization procedure to ensure stable baseline performance before proceeding with the exposure to trunk flexion. It could be that previous results regarding sitting postural sway (Hendershot et al., 2013) were confounded by poorer reliability, although learning effects would work in the opposite direction.

Despite the factors discussed above, a complete lack of effect of prolonged intermittent flexion on sitting postural control is surprising, considering that previous studies have shown alterations in intrinsic trunk stiffness and reflex behavior (Granata et al., 2005b; Rogers and Granata, 2006; Sánchez-Zuriaga et al., 2010; Bazrgari et al., 2011; Hendershot et al., 2011; Voglar et al., 2016). It seems that the body can successfully adapt to effects of fatigue by increasing background muscle activation (Grondin and Potvin, 2009) and possibly by increasing reflex gains (Hendershot et al., 2011). Moreover, our previous finding of increased reflex gains alongside significant creep deformation, supports the assumption that the body can successfully compensate for decreased passive stiffness (Voglar et al., 2016). As trunk muscle activation is increased when the sitting surface becomes less stable (Oomen et al., 2015), such an increase in muscle activation likely enhances stabilization. Importantly, the changes in reflex behavior following prolonged flexion seem to be driven primarily by creep deformation, and to a lesser extent by muscle fatigue (Sánchez-Zuriaga et al., 2010; Voglar et al., 2016). However, postural sway is known to be heavily influenced by both local and global muscular fatigue (Springer and Pincivero, 2009; Paillard, 2012; Van Dieën et al., 2012; Garcia-Gallart and Encarnacion-Martinez, 2019), as well as cognitive fatigue (Noé et al., 2021). Therefore, monitoring muscular and cognitive fatigue during/after exposure is recommended for easier interpretation of results pertaining to CoP movements. Despite the fact that lumbar muscles were more active in the unsupported condition (Voglar et al., 2016), the resulting fatigue, if any, did not affect CoP behavior during sitting.

### Limitations

There are a few limitations of the present study that need to be acknowledged. Firstly, a convenience sample of young healthy adults was used. It could be that different responses would be seen in participants of different ages, LBP history, and professions. Next, the sample size of the study was relatively small. It could be that differences in CoP variables would be seen if larger sample was used. Given the high variability across participants, a very large sample size would likely be needed. Note that effects would be small relative to this variability and hence likely not important. While the protocol was well-controlled, performing a task that would simulate actual work activities (e.g., crane operator shift) would increase the ecological validity of the study. Despite providing extensive familiarization and thus high reliability, another limitation of our measurements is a rather high between-participant variability, with coefficients of variation ranging from 21.4 to 51.3%. This could mask small changes induced by exposure to prolonged trunk flexion. Moreover, postural stability is a phenomenon with many underlying factors, including sensory contributions from vestibular, visual and somatosensory systems (Horak et al., 2009), sensory integration (Goodworth and Peterka, 2009), reflex behavior (Chen and Zhou, 2011), and joint stiffness (Sakanaka et al., 2021), contributions of which are in part modulated to deal with surface instability (Andreopoulou et al., 2015) and other task constraints (van Drunen et al., 2015). Moreover, small increases in CoP movements are not necessarily indicative of deteriorated balance, but may simply reflect altered balance maintained strategy, which may not be related to injury risk (Shumway-Cook and Woollacott, 2017). Somewhat different responses across participants (Figure 3) could indicate that they used different strategies to adapt to the changes induced by the exposure to flexion. It is not clear whether small increases in CoP movement actually reflect decreased stability during sitting. It is suggested that future studies explore the effects of trunk flexion on postural control in more detail. For instance, it would be interesting to explore specific strategies used to maintain trunk stability during sitting. In LBP patients, an increased reliance on co-contraction and lower reliance on cognitive control for static standing posture has been reported (Kiers et al., 2015). Co-contraction may not be an optimal strategy to maintain balance, as it is associated with increased spinal compressive forces (Granata et al., 2005a).

## Conclusion

In conclusion, regardless of the presence of a trunk support, prolonged intermittent flexion did not induce any changes in CoP behavior during a seated balance task. This suggests a successful compensation of decreased passive stiffness by increased reflex activity. Future studies should assess the effects of longer exposures to flexion on postural control, possibly simulating real-life work shifts (e.g., crane operator shift).

## Data Availability Statement

The raw data supporting the conclusions of this article will be made available by the authors, without undue reservation.

## Ethics Statement

The studies involving human participants were reviewed and approved by Ethics committee for Movement Sciences (Ethische Commissie Bewegingswetenschappen) at the Vrije Universiteit, Amsterdam. The patients/participants provided their written informed consent to participate in this study.

## Author Contributions

MV, IK, JD, and NŠ conceptualized the idea. MV carried out the measurements. JD and NŠ were overviewing the measurement procedures and administration. MV and ŽK analyzed the collected data and wrote the first draft of the manuscript. All authors worked on finalizing the manuscript and approved the submitted version.

## Conflict of Interest

NŠ was employed by company S2P, Science to Practice, Ltd. The company had no role in conceptualization of the study, data acquisition, article writing, nor any other phase of the study. The remaining authors declare that the research was conducted in the absence of any commercial or financial relationships that could be construed as a potential conflict of interest.

## Publisher’s Note

All claims expressed in this article are solely those of the authors and do not necessarily represent those of their affiliated organizations, or those of the publisher, the editors and the reviewers. Any product that may be evaluated in this article, or claim that may be made by its manufacturer, is not guaranteed or endorsed by the publisher.
